# Association of sleep quality with excessive daytime somnolence and quality of life of elderlies of community

**DOI:** 10.1186/s40248-018-0120-0

**Published:** 2018-03-15

**Authors:** Glauber Sá Brandão, Fernanda Warken Rosa Camelier, Antônia Adonis Callou Sampaio, Glaudson Sá Brandão, Anderson Soares Silva, Glaucia Sá Brandão Freitas Gomes, Claudio F. Donner, Luis Vicente Franco Oliveira, Aquiles Assunção Camelier

**Affiliations:** 1 Postgraduate Program in Medicine and Human Health, School of Medicine and Public Health in Bahia, Salvador, BA Brazil; 2 Department of Education (DEDC-VII), University of the State of Bahia – UNEB, Rodovia Lomanto Júnior, BR 407, Km127, Senhor do Bonfim, BA CEP 48970-000 Brazil; 3 Department of Life Sciences (DCV), University of the State of Bahia - UNEB, Salvador, BA Brazil; 4 Diagnostic and specialty clinic (IMAIS), Senhor do Bonfim, BA Brazil; 5 Sleep Laboratory, Rehabilitation Sciences Master’s and PhD Degree Program, Nove de Julho University (UNINOVE), Sao Paulo, SP Brazil; 6 Mondo Medico, Multidisciplinary & Rehabilitation Outpatient Clinic, Borgomanero, NO Italy; 7 Medical School , University Center of Anapolis – UniEVANGELICA, Anapolis, GO Brazil

**Keywords:** Elderly, Community, Sleep, Quality of life, Excessive daytime somnolence

## Abstract

**Background:**

The progressive increase in the elderly population contributes to the fact that studies on human aging have important attention of health professionals and government agents, since they present a great challenge regarding public health. Our objective is to characterize the profile of older people with poor sleep quality and analyze possible associations with excessive daytime somnolence, quality of life and functional mobility.

**Methods:**

This is a cross-sectional descriptive study, involving elderlies of the community, with the questionnaires Pittsburgh Sleep Quality Index (PSQI), Epworth Sleepiness Scale, WHOQOL-OLD and application of the Timed Up and Go test - TUG. Descriptive statistics, Student’s t test for paired samples and Pearson’s correlation coefficient (*p* ≤ 0.05) were used.

**Results:**

We recruited 131 elderly people, predominantly female (87%); mean age 68 ± 7 years, low *per capita *income (84.8% ≤ 2 minimum wage), low education (86.3% ≤ 3 years of study), and mostly staying with relatives (67.9%), married (39.7%) or amassed (35.9%). Seventy-one percent of the sample is above normal weight, 90.1% of women have an abdominal circumference ≥ 80 cm and a high prevalence of chronic and psychosocial diseases was identified in the self-report, and the risk of obstructive sleep apnea in 38.2%. The mean PSQI, Epworth Sleepiness Scale, WHOQOL-OLD and TUG were equal to, respectively, 11.2 ± 3.2; 8.32 ± 2.2; 84.8 ± 10.2 and 8.97 ± 2. An association of sleep quality with excessive daytime somnolence and quality of life, while not with functional mobility, was observed.

**Conclusion:**

The results of the present study allowed to identify a sleep quality associated with excessive daytime somnolence and quality of life and also to characterize the profile of elders with poor sleep quality.

## Background

The elderly population has the highest growth rate in the world. In socioeconomic developing countries such as Brazil, population aging is a relatively new process and has occurred so rapidly that the more conservative projections indicate that by 2025 it will be the sixth country in the world in terms of the number of elderly people, corresponding to 15% of all the population contingent [1]. This progressive increase in the elderly population contributes to the fact that studies on human aging have important attention of health professionals and government agents, since they present a great challenge regarding public health [2].

The physiological aging is accompanied by alterations in the quality, quantity and architecture of the sleep, being able to lead to diverse diseases, originating considerable social and economic problems [3–6].

Studies using electroencephalography have shown that the cortical status of older people presents a tendency to alertness, associated with a low threshold to wake up in response to environmental stimuli and an increased sleep latency. Thus, a reduction in the ability of healthy elderly individuals to initiate and maintain sleep associated with an increase in stages 1 and 2 and a reduction of stage 3 of NREM sleep is observed, as well as a reduction of REM sleep [4, 6].

This reduction of slow-wave sleep may be a consequence of the loss of the predominance of parasympathetic activity, as evidenced by the reduction of heart rate variability during sleep in the elderly, and results in a decline in the secretion of human growth hormone, thus reducing restorative capacity [5, 7, 8].

Among the several diseases that affect the elderly, sleep disorders are among the most prevalent and they are associated with several comorbidities, which consist by a relevant factor of alterations in the perception of general health [9, 10]. An important epidemiological study with more than 9,000 participants has demonstrated that more than half of the elderly population had at least one complaint of sleep and that the poor quality of sleep would be associated with the presence of biopsychosocial deficiencies such as excessive daytime somnolence, cognitive deficit, depression, fatigue, increased risk of falls, limitations in daily life activities, reduced quality of life and increased incidence of cardiovascular morbidity and mortality [11].

According to the high prevalence of sleep disorders in the geriatric population, we highlight the necessity for information which may subsidize health services in the planning of care in relation to sleep quality and its health impacts in elderly. Therefore, the present study aimed to test the association of sleep quality with excessive daytime somnolence, quality of life and functional mobility of the elderly with poor sleep quality.

## Methods

This is a descriptive, cross-sectional study with a quantitative approach on the profile of the elderly in the community with poor sleep quality, approved by the Research Ethics Committee of the Bahia School of Medicine and Public Health (EBMSP), with CAAE: 39072514.6.0000.5544. All participants in the study signed the Informed Consent Term. The design and conduct of this study followed the guidelines of the Reporting of Observational Studies in Epidemiology (STROBE).

The study was conducted with elderly residents in Senhor do Bonfim-BA, northeast region of Brazil, from July to November 2015. Recruitment occurred throughout the community, through the dissemination of study in local newspapers, radios, religious centers, groups of elderly meeting, senior residence, neighborhood association and in the third age project developed by the city hall, and in the announcement the phone number was provided for interested parties in order to contact the researchers. The inclusion criteria were 60 years old or older and score ≥ 5 in the Pittsburgh Sleep Quality Index (PSQI) [12]. Participants with a cognitive decline according to the Mental State Mini-Exam [13] and those who were performing some treatment for sleep disorders were excluded from the study.

A questionnaire was applied covering socioeconomic and demographic variables, as well as questions regarding self-reported morbidities and life habits.

Sleep quality was assessed using the Pittsburgh Sleep Quality Index (PSQI) validated for using in Brazil [12]. The PSQI was developed in 1989 and provides a measure of quality of standardized sleep, which discriminates the participants in good or bad sleepers. It consists of seven components, each scored on a scale of zero to three. The components are, respectively; sleep subjective quality; sleep latency; duration of sleep; habitual sleep efficiency; sleep disturbances; using medications for sleep and daytime dysfunction. The scores of the seven components are added together to give an overall score ranging from 0 to 21, with scores of 0–4 indicating good quality of sleep, 5–10 poor quality and above 10 sleep disorder [14].

Excessive daytime somnolence was assessed using the Epworth Sleepiness Scale (ESS), validated for Brazil. The scale presents eight situations involving activities of daily living in the occurrence of daytime sleepiness. The participants were instructed to rate on a score from 0 to 3 about the probability of feeling slumber or falling asleep in each of the eight specific situations, in which the higher scores indicate a greater chance of sleeping and scores above 10 suggesting a diagnosis of excessive daytime somnolence [15].

To evaluate the potential risk of Obstructive Sleep Apnea (OSA), the Berlin Clinical Questionnaire was used. The instrument includes ten items organized in three categories: snoring and apnea (containing 5 items), daytime somnolence (4 items), systemic arterial hypertension and obesity (1 item). The categories 1 and 2 are considered positive if the score of each is greater than or equal to two points, while category 3 is considered positive if the answer to question 10 is YES or if the body mass index (BMI) is greater than 30 kg/m^2^. Patients are considered to be at high risk for OSA, when two or more categories present a positive score and, when there is none or only one category with a positive score, the risk for OSA is low [16].

In evaluating the anthropometric variables such as body weight and height, a balance with estadiometer was used (mechanical anthropometric scale, 150 kg - Welmy®, Sao Paulo, Brazil) properly calibrated; to measure the waist circumference, a tape measure was used and the Body Mass Index (BMI) was calculated from the weight in kilograms divided by the height in meters squared.

The level of physical activity was evaluated through the International Questionnaire of Physical Activity (IPAQ) adapted for the elderly [17]. It is an instrument which allows estimating the weekly energy expenditure of physical activities related to work, transportation, domestic tasks and leisure, carried out continuously for at least 10 min with moderate and/or vigorous intensity during a normal/usual week. For the present study, the last variable was dichotomized, and it was considered inactive that participant who executed less than 150 min per week of moderate and/or vigorous and active that participant who executed physical activities for more than 150 min per week.

Functional mobility was assessed using the *Timed Up and Go test* (TUG), a simple test which evaluates the execution speed in getting up from a chair with arms, to walk three meters forward, to turn around, to walk back and to sit on the chair again. The execution time less than 10 s suggests totally free and independent individual; those who perform the test between 10 and 19 s are considered independent, from 20 to 29 s those who are in a so-called “gray zone”, which is, they demonstrate difficulties for tasks of daily living and limited functional capacity. Those who present a time score of 30 or more seconds tend to be totally dependent on many basic and instrumental activities of daily living [18].

To evaluate the quality of life used the WHOQOL-OLD containing six facets each of 4 items, which was evaluated by *Likert scale* (1 to 5 points): Facet I **-** “Sensory Operation”; Facet II **-** “Autonomy”; Facet III **-** " Past, Present and Future Activities"; Facet IV **-** “Social Participation”; Facet V **-** “Death and Dying”; Facet VI **-** “Intimacy”. Each of the facets has 4 items, thus for all facets the score of the possible values may range from 4 to 20, and the scores of these six facets or the values of the 24 items may be combined to produce a “global” score of the quality of life in the elderly and the higher the score the better the quality of life [19].

The data were tested for normality *(Kolmogorov-Smirnov test)* were subjected to a descriptive analysis by the absolute frequencies and percentages for categorical variables and measures of central tendency and dispersion for numerical variables. It was used the Student’s T test for comparison of means, and the graphical representation of the box diagram *(boxplot)* to demonstrate the behavior of continuous variables between groups. The Pearson correlation coefficient (r) was also used to analyze the association of PSQI with the following variables: ESE, TUG and WHOQOL-OLD. For decision criteria, it was adopted the significance level of 5% (*p* ≤ 0.05) and statistical procedures were processed and analyzed in *Statistical Package of the Social Sciences* (SPSS) for Windows®, version 21.

## Results

**Fig. 1:**
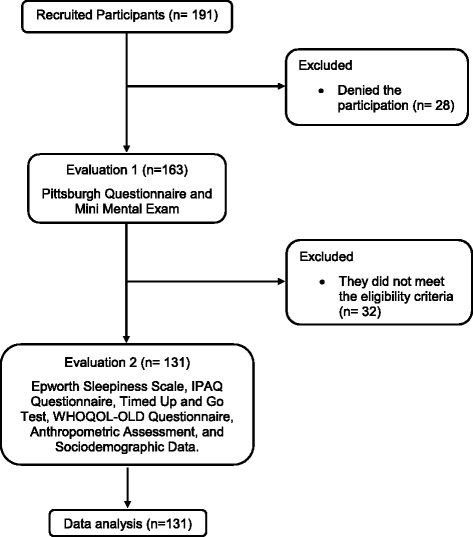
Flowchart of the study

One hundred and ninety-one potential participants were recruited from the community. Twenty-eight refused to participate in the study and 32 were excluded according to the eligibility criteria, which left only 131 participants, which represented 80.4% of the sample. A summary of participants’ flow over the course of the study is presented in Fig. 1.

Table 1 shows the predominance of females (87%); with a mean age of 68 ± 7 years, the majority being between 60 and 69 years of age. This is a population predominantly low income *per capita* (84.8% ≤ 2 minimum wage), low education (86.3% ≤ 3 years of study) and, mostly living with relatives (67.9%), married (39.7%) or cohabiting (35.9%). Regarding anthropometry, 47.3% of the elderly were overweight and 23.7% were obese, which is associated with the fact that 90.1% of the women had abdominal circumference ≥ 80 cm.

**Table 1:** Sociodemographic and anthropometric characteristics of the interviewed elderly

	Total (*n* = 131)	
Variables	N	%
Gender		
Female	114	87
Male	17	13
Age (years)		
60 to 69	68	51.9
70 to 70	48	36.6
≥ 80	15	11.5
Education		
Illiterate	14	10.7
1 to 3 years	99	75.6
4 to 7 years	15	10.7
8 years or more	3	2.3
Monthly income per capita		
< 1 MW	58	44.3
1 to 2 MW	53	40.5
> 2 to 3 MW	12	9.2
> 3 MW	8	6.1
Family composition		
Live alone	42	32.1
Live with relatives	89	67.9
Marital status		
Single	11	8.4
Married	52	39.7
Widowed	8	6.1
Cohabiting	47	35.9
Divorced	13	9.9
BMI (Kg/m^2^)		
Low weight (< 18.5)	4	3.1
Normal (18.5 to 24.9)	34	26.0
Overweight (25.0 to 29.9)	62	47.3
Obese (≥ 30)	31	23.7
Waist Circumference (cm)		
Women		
Increased risk^a^ (WC ≥ 80 cm)	118	90.1
Men		
Increased risk^a^ (WC ≥ 94 cm)	2	1.5

*MW* minimum wage during the study (in real) = R$ 788.00, *BMI* Body mass index, *WC* Waist Circumference

^a^Risk for cardiovascular disease

Among the self-reported morbidities, anxiety (58%), arthrosis (37.4%), systemic arterial hypertension (33.6%), diabetes (26%) and chronic pain were the most prevalent (38.9%). Regarding lifestyle habits, 90.1% of the elderly reported not smoking, and out of these non-smokers, 12.2% reported smoking in the past. Most participants did not report alcohol consumption and presented a level of physical activity considered active (> 150 min/week), according to the IPAQ (Table 2).

**Table 2:** Self-reported morbidities, life habits and physical activity level of the interviewed elderly

	Total (*n* = 131)	
Variables	N	%
Morbidities		
Diabetes	34	26.0
Systemic arterial hypertension	44	33.6
Urinary incontinence	32	24.4
Stroke	6	4.6
Respiratory disease	14	10.7
Arthritis	24	18.3
Arthrosis	49	37.4
Fibromyalgia	6	4.6
Depression	29	22.1
Anxiety	77	58.8
Chronic pain	51	38.9
Smoking		
Yes	13	9.9
No	118	90.1
Ex-smokers	14	12.2
Alcohol use		
Yes	17	13.0
No	114	87.0
Physical Activity Level – IPAQ		
Inactive (< 150 min/week)	17	13
Active (> 150 min/week)	114	87

Data regarding sleep quality, daytime somnolence symptoms, quality of life and functional mobility (TUG) are described in Table 3.

**Table 3:** Mean values and standard deviation for the PSQI, ESE, WHOQOL-OLD, and TUG performance time in the elderly community (*n* = 131)

Variables	Mean ± SD	Variation
Sleep Quality (PSQI)	11.2 ± 5.6	5–18
Excessive daytime somnolence (ESS)	8.3± 2.2	4–14
Quality of life (WHOQOL-OLD)	84.8 ± 10	62–111
Functional mobility (TUG)	9.0 ± 2.0	5–14

From the 131 elderlies with poor sleep quality, 76 (58%) had sleep disorder (PSQI score >  10 points), 40 (30.5%) had excessive daytime somnolence assessed by ESS, and 50 (38.2%) were considered to be at high risk for OSA because they presented positive scores in two or more categories of the Berlin Clinical Questionnaire. The average nightly sleep time was 5.7 ± 0.94 h and 55 elderlies (42%) reported waking up at night to go to the bathroom. In Table 4 we can see the comparison of baseline characteristics by Pittsburgh Sleep Quality Index.

**Table 4:** Comparison of baseline characteristics by Pittsburgh Sleep Quality Index

Variables	5 a 10	> 10	*p*
Gender (% women)	50	64	0.26
Age (years)	71.2 ± 6.2	69.8 ± 7.5	0.07
BMI (Kg/m^2^)	27.3 ± 4.2	27.2 ± 4.1	0.95
Waist Circumference (cm)	92.8 ± 10.6	92.4 ± 10.3	0.82
Self-reported morbidities (n)	1.73 ± 1.2	1.82 ± 1.7	0.74
Per capita income (% ≤ 2 minimum wages)	43	55	0.95
Education (% ≤ 3 years of study)	45	62	0.28
Housing (% live with relatives)	42	47	0.08

Table 5 and Table 6 show the comparison of the questionnaire scores according to the gender and BMI of the subjects involved in this study.

**Table 5:** Comparison of the questionnaires scores according to gender

Variables	Female	Male	*p*
PSQI	11.2 ± 3.3	11.6 ± 2.5	0.6
ESS	8.3 ± 2.2	8.2 ± 1.9	0.8
WHOQOL-old	84.4 ± 10.4	83.9 ± 10.6	0.8
BQ (% high risk)	38	34	0.2
IPAQ (% actives)	85	90	0.1

*PSQI* Pittsburgh Sleep Quality Index, *ESS* Epworth Sleepiness Scale, *BQ* Berlin Questionnaire, *IPAQ* International Physical Activity Questionnaire

**Table 6:** Comparison of the questionnaire scores according to Body Mass Index

Questionnaires	Normal weight	Overweight	Obese	*p*
PSQI	11.21 ± 3.7	11.18 ± 3	11.34 ± 3	0.97
ESS	7.94 ± 2.4	8.37 ± 2.2	8.66 ± 2.1	0.43
BQ (% high risk)	37	36	38	0.13
IPAQ (% actives)	89	87	86	0.24

*PSQI* Pittsburgh Sleep Quality Index, *ESS* Epworth Sleepiness Scale, *BQ* Berlin Questionnaire, *IPAQ* International Physical Activity Questionnaire

**Fig. 2:**
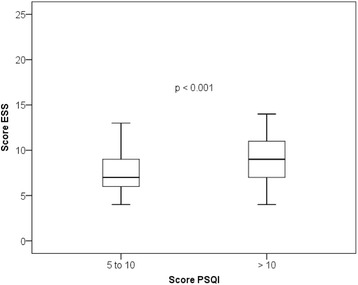
Distribution boxplot graph ESS score of the two groups with different scores in PSQI

After dividing the sample into two groups with different PSQI scores (5 to 10 versus > 10 points) and performing Student’s t-test to compare ESS means between groups (*p* < 0.01), it was represented, in the Fig. 2, the ESS score distribution for each group using the boxplot graph*,* with a median 7 (4–13) for the group (5 to 10), median 9 (4–14) the group (> 10). The correlation of the PSQI score with ESS was moderate and statistically significant (*r* = 0.4 and < 0.001).

**Fig. 3:**
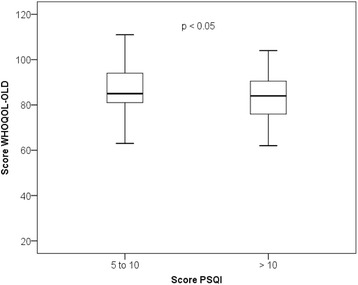
Distribution boxplot graph of WHOQOL-OLD score in relation to two subgroups with different scores in PSQI

By using the same groups of the PSQI (5 to 10 versus > 10 points), and after carrying out the Student’s t test to compare the WHOQOL-OLD means between the groups (p < 0.01), it was represented in Fig. 3, the WHOQOL-OLD score for each group, with a median of 85 (63–111) for the subgroup (5 to 10) and a median of 84 (62–104) for the subgroup (> 10). An association between PSQI and WHOQOL-OLD scores (*r* = − 0.285 and p < 0.01) was identified.

Regarding the TUG, the PSQI group (5 to 10) had a median 8.5 (5–14) and the group with PSQI (> 10) had a median 8.9 (5.7–14), no difference between groups and a weak association were found, with no statistical significance between TUG and IPAQ (*r* = − 0.13 and *p* = 0.89).

## Discussion

In the sample of patients in the present study, we observed the predominance of female gender (87%), reflecting the greater longevity of women in history and worldwide. The great number of females in old age is associated with several aspects, such as less exposure to occupational risk factors, lower prevalence of smoking and alcoholism, greater attention to health and self-care, and greater frequency of using the health services [20, 21].

There was a preponderance of 60 to 69 years of age (51.9%), with a mean age 68 ± 7 years, converging with international [22] and national studies [23], positively qualifying the external validity of the present study. This preeminence of age at the lower limit is evidence of the recent aging of the Brazilian population, unlike the developed countries, where the elderly is aged 80 and over [24].

In the present study, 75.6% of the elderly have low educational level, reflecting the intense socioeconomic inequality experienced in the country [25, 26]. In addition, a low economic status was identified, with 44.3% presenting a family income below 1 minimum wage, converging with epidemiological profile studies conducted in Brazilian cities [26]. Despite these socioeconomic and demographic characteristics, it was possible to properly apply the questionnaires and extract important information by analyzing the results, encouraging further studies.

In the anthropometric analysis, the BMI indicates that 47.3% are overweight and 23.7% are obese, which is, 71% of the interviewed elderly are overweight considered adequate [27]. These weight changes associated with the fact that 90.1% of the women present waist circumference ≥ 80 cm, present a high risk of cardiovascular diseases [28] and the Obstructive Sleep Apnea Syndrome, possibly related to pharyngeal narrowing by fat deposition in its walls or the parapharyngeal structures [29]. On the other hand, growing evidence indicates that short sleep duration, as presented in the present study, avoiding a possible selection bias, the PSQI, ESS, WHOQOL-old, Berlin Questionnaire and IPAQ questionnaire scores were compared according to the gender and also according to the BMI of the subjects involved in the study. No significant difference was observed.

Where the average nightly sleep time was 5.7 ± 0.94 h, it is considered a risk factor for the development of obesity and its subsequent complications [30, 31]. Sleep loss may lead to metabolic and endocrine disorders, including reduced glucose tolerance and changes in appetite-regulating hormones, such as the ghrelin, a hunger-promoting hormone which increases with sleep restriction, while leptin is the hormone which contributes to satiety is reduced [29, 30].

Associated with the growing process of population aging, functional changes occur which are characteristic of this age group, predisposing to the appearance of pathologies classified as chronic non-transmissible [32]. The present study found a high frequency of elderly patients with self-reported chronic and psychosocial disorders, highlighting the anxiety, followed by chronic pain, osteoarthritis, hypertension, and diabetes*,* as observed in epidemiological studies on aging [33, 34]. The chronic and psychosocial diseases are negatively associated with self-perception of health, quality of life and sleep quality of the elderly [32]. This panorama points to the real necessity to strengthen the health promotion actions.

Regarding lifestyle habits, few elderly people reported smoking and alcohol consumption, which may be explained by the predominance of females in the sample, considering that women devote more attention to health and self-care [20, 21]. According to the IPAQ, most of participants were considered active, diverging from other studies in which the level of physical activity is presented inversely proportional to income and education [33, 34].

In evaluating the quality of nocturnal sleep, the sample showed an overall average score 11.2 ± 5.6 in the PSQI, classified as sleep disorder. This result is similar to that presented by other studies [35, 36]. Among the problems related to nocturnal sleep, we highlight the high risk of OSA in 38.2% of the sample, corroborating the important epidemiological study where it was found that the incidence of OSA is proportional to age, female gender and low socioeconomic class and presents important interference in sleep quality [37]. It is worth noting the necessity to wake up to go to the bathroom, reported by 42%, which may also interfere with sleep quality [38].

As for excessive daytime sleepiness, 40 elderly participants (30.5%) had a score ≥ 10 in ESS, however the mean score of the sample was 8.32 ± 2.2, which is not indicative of excessive daytime somnolence, converging with previous studies [36, 39]. Excessive diurnal somnolence reverberates in the life of the elderly predisposing to naps that, when prolonged, may be harmful because they interfere in the duration and quality of nocturnal sleep [38]. In the present study, the daytime somnolence is directly associated with worse perception of nocturnal sleep quality, corroborating previous investigations [40, 41]. The nocturnal sleep restriction, identified in our sample through PSQI (nocturnal sleep duration 5.7 ± 0.94), may result in a sensation of physical and mental fatigue during the day, predisposing to excessive daytime sleepiness [42].

The quality of life of the interviewed elderly was considered good, with a mean score in the WHOQOL-OLD 84.8 ± 10, similar to that found in another study which has compared the quality of life among the young and very old elders and obtained a total score 84.1 ± 11.5 and 83.3 ± 12.3, considered as good quality of life for both groups [43]. In the present study, it was found that the deterioration of sleep quality presents a significant but weak association, with the deterioration in the perception of quality of life, in consonance with results presented in other studies [44]. Sleep deprivation may adversely affect the quality of life of elderly by altering proprioception, neuromuscular reaction time and postural control, reducing independence and autonomy [45]; increasing pain sensitivity [46]; triggering neurobehavioral impairments such as decline in attention and memory and it may be a risk factor for the development of stress, anxiety and depression [47].

To move from sitting to standing position is an activity considered to be complex and most performed in the daily life of the elderly, and it is important in the evaluation of functional decline, which occurs proportionally as the age advances [48]. Functional mobility was assessed through the TUG and obtained a mean score 8.9 ± 2, suggestive of totally unrestricted, independent elderly and classified as low risk for falls [18]. According to the results presented, it is possible to hypothesize that sleep disturbance is not associated with changes in functional mobility and risk of falls in the elderly, diverging from previous studies that obtained this association [49], however, we cannot exclude the possibility of type II statistical error. These aspects may be evaluated in future studies specially designed for this purpose.

The study presents some limitations that must be considered. The cross-sectional design shows the reverse causality bias, since it is not possible to obtain information regarding the natural history of the diseases and/or events; the study recruitment was directed at older adults in the general community who presented self-reported sleep complaints but the results may be more clinically significant if the recruitment is performed in the elderly with clinical diagnosis of sleep disturbance through polysomnography or polygraphy; The questionnaires were used in the elderly in different degrees of education, which may interfere in the quality of the answers, but all the subjects who presented difficulties in answering the questionnaires were assisted; only the elderly with a PSQI score ≥ 5 were studied, and the elderly with good sleep quality were not studied.

## Conclusion

Among the elderly in the community selected with PSQI scores ≥5, the poor quality of nocturnal sleep is associated with worse quality of life and the presence of symptoms of excessive daytime somnolence, however not significantly influencing functional mobility.

## Abbreviations

BMI: Body mass index
ESS: Epworth Sleepiness Scale
IPAQ: International Questionnaire of Physical Activity
OSA: Obstructive Sleep Apnea
PSQI: Pittsburgh Sleep Quality Index
STROBE: Reporting of Observational Studies in Epidemiology
TUG: Timed Up and Go test
WHOQOL-OLD: World Health Organization Quality of Life for Older Adults
